# An Immunofluorescence-Assisted Microfluidic Single Cell Quantitative Reverse Transcription Polymerase Chain Reaction Analysis of Tumour Cells Separated from Blood

**DOI:** 10.5772/61822

**Published:** 2015-11-02

**Authors:** Kazunori Hoshino, HaeWon Chung, Chun-Hsien Wu, Kaarthik Rajendran, Yu-Yen Huang, Peng Chen, Konstantin V. Sokolov, Jonghwan Kim, John X.J. Zhang

**Affiliations:** 1 Department of Biomedical Engineering, University of Connecticut, Storrs, CT, USA; 2 Department of Molecular Biosciences, Center for Systems and Synthetic Biology, University of Texas at Austin, Austin, TX, USA; 3 Department of Biomedical Engineering, University of Texas at Austin, Austin, TX, USA; 4 Department of Imaging Physics, University of Texas MD Anderson Cancer Center, Houston, TX, USA; 5 Thayer School of Engineering, Dartmouth College, Hanover, NH, USA

**Keywords:** CTC, single cell PCR, breast cancer, immunomagnetic assay, lab on a chip, laser microdissection

## Abstract

Circulating tumour cells (CTCs) are important indicators of metastatic cancer and may provide critical information for individualized treatment. As CTCs are usually very rare, the techniques to obtain information from very small numbers of cells are crucial. Here, we propose a method to perform a single cell quantitative reverse transcription polymerase chain reaction (qPCR) analysis of rare tumour cells. We utilized a microfluidic immunomagnetic assay to separate cancer cells from blood. A combination of detailed immunofluorescence and laser microdissection enabled the precise selection of individual cells. Cancer cells that were spiked into blood were successfully separated and picked up for a single cell PCR analysis. The breast cancer cell lines MCF7, SKBR3 and MDAMB231 were tested with 10 different genes. The result of the single cell analysis matched the results from a few thousand cells. Some markers (e.g., ER, HER2) that are commonly used for cancer identification showed relatively large deviations in expression levels. However, others (e.g., GRB7) showed deviations that are small enough to supplement single cell disease profiling.

## 1. Introduction

Circulating tumour cells are cells that have detached from a primary tumour and circulate in the blood stream[1, 2]. The detection and analysis of CTCs has been an object of interest because simple blood testing may indicate disease activity, provide evidence of progressive disease and diagnose cancer earlier. Several approaches, including the commercially available CellSearch™ method [3, 4], have been proposed and shown some success for the enumeration of CTCs in blood. However, techniques that allow for a detailed genomic and immunofluorescence analysis of separated cancer cells rather than a simple enumeration are needed to exploit the potentially abundant information that we may obtain from the CTC.

The polymerase chain reaction (PCR) is among the most widely utilized techniques for the genomic analysis of tumours [5]. A PCR analysis of CTCs is advantageous because it may provide genomic information of tumours that would otherwise be obtained from an invasive tumour biopsy. An analysis of cancer cells from blood is challenging because of the low cancer cell-blood cell ratio, which could be as low as 1 to 10^7^. It is also challenging because, in many cases, the number of cancer cells found in 5-10 mL of a patient's blood is less than five. Approaches to carrying out a PCR analysis for CTCs have been reported and some allow for a single cell analysis [6][Bibr bibr7-61822][Bibr bibr8-61822][Bibr bibr9-61822][Bibr bibr10-61822]–[11]. However, reliable techniques to study genomic information of individual CTCs, which are also characterized by detailed immunofluorescence, are still to be studied. Conventional tools for fluorescence-activated cell sorting [12][Bibr bibr13-61822]–[14] (FACS), such as the BD FACSAria™, rely on cell fluorescence intensities but are more suitable for sorting or enriching larger numbers of target cells. In a CTC study, a detailed microscopic observation of cell morphology, along with an immunofluorescence observation, is crucial to correctly identify the very rare cancer cells. For a single cell PCR analysis, sample purity is also a critical parameter. In order to discuss the characteristics of the individual cancer cells that are of interest, other blood cells should be eliminated from the sample. A separation technique that is based on a detailed immunofluorescence observation is needed for a single cell PCR analysis of CTCs.

In this paper, we propose a method for a single cell PCR analysis that is based on the microfluidic immunomagnetic separation of cancer cells. A combination of a microscopic fluorescence observation and the laser microdissection technique [15] allows for the accurate separation of cancer cells for the following single cell PCR analysis. In addition, the collected cancer cells are fixed on standard microscope glass slides and many existing microscopic observation techniques can be used before carrying out a single cell PCR analysis.

## 2. Results

### 2.1 Microchip-based Immunomagnetic Separation

Blood samples were drawn from a healthy donor using CellSave™ tubes (Janssen Diagnostics, LLC, NJ). The samples were then added with control breast cancer cell lines. As model breast cancer cell lines for ER/PR positive, HER2 positive and triple negative breast cancers, we used MCF7, SKBR3 and MDAMB231 cell lines [16], respectively. The cultured cells were harvested, centrifuged and resuspended in buffer solution. The cells were then counted with a haemocytometer and diluted in phosphate buffered saline (PBS) to prepare a solution with a known concentration of suspended cells. The number of cancer cells that were added in 2.5 mL of the samples ranged from 5-1000. Before microfluidic screening, the blood plasma was replaced with a buffer and the red blood cells (RBCs) were lysed and washed in order to reduce potential contaminants for the single cell PCR. We centrifuged 2.5 mL aliquot of blood and replaced the plasma with a buffer solution (Veridex, LLC, NJ) to obtain a total of 3.5 mL of blood solution. This was then added with a 1X lysis buffer (BioLegend, CA). After 10 minutes of incubation, the mixture was centrifuged for 5 minutes at 300 g (relative centrifugal force). The supernatant was then aspirated and the cell pellet was resuspended in a buffer solution (Veridex, LLC, NJ).

**Figure 1.**
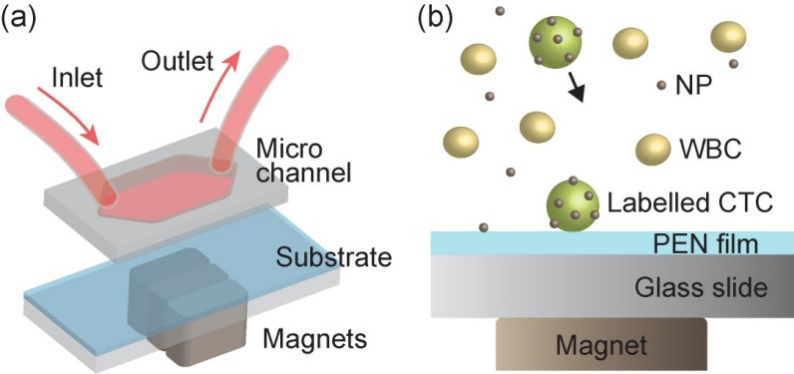
The experimental setup of the microfluidic immunomagnetic assay. The cancer cells labelled with EPCAM functionalized the magnetic nanoparticles, which were attracted onto a PEN film-coated glass slide.

The details of our microchip-based separation method have been reported elsewhere [17]. The blood samples were added with a suspension of Fe_3_O_4_ magnetic nanoparticles (size: 100-200 nm), functionalized with anti-epithelial cell adhesion molecules (anti-EpCAM). The microchip-based screening process started after 10 minutes of incubation. Figure 1 shows diagrams of the experimental setup. The cancer cells that were bound to the particles were collected onto glass slides by permanent magnets as the blood flowed through the microchip. On top of the glass substrates was a 3-μm-thick polyethylene naphthalate (PEN) film. We used PEN film coated glass slides (Membrane Slide 1.0 PEN), which were supplied by Carl Zeiss Ltd. The surface of the PEN film was treated with a 0.01% (w/v) solution of poly-L-lysine (Sigma). After blood screening, a buffer solution was introduced to flush the unwanted white blood cells (WBCs). The capture rate of the microfluidic system was defined as the number of cancer cells that were found on the slide, divided by the number of cells that were added to the blood sample. Details of the capture efficiency that were tested with the spiked samples and patient samples have been reported elsewhere [17][Bibr bibr18-61822]–[19]. For the spiked experiments with SKBR3 and MCF7 cells, the capture rates were consistently better than 90%. From the patient samples, the number of cancer cells that were typically found was 1-10. We have also seen previous cases with more than 1000 cells in 2.5mL of blood [18].

### 2.2 Immunofluorescence-assisted Cell Identification and Separation

**Figure 2.**
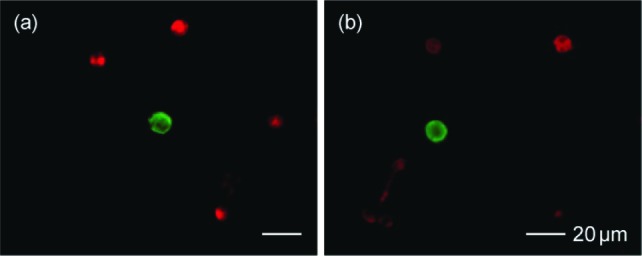
The captured breast cancer cells of (a) SKBR3 and (b) MCF7 stained with FITC (green). Non-specifically captured WBCs stained with AlexaFluor 594 (red) are also found in the images. Separate photographs that were taken with green and red filters are shown as overlay images.

The cells that were captured on the slide were fixed using acetone and fluorescently stained with anti-cytokeratin (mouse anti-cytokeratin, pan-FITC, Sigma-Aldrich, St. Louis, MO) and anti-CD45 (AlexaFluor 594, Invitrogen, Carlsbad, CA, bound to mouse anti-human clone 9.1) for the positive and negative tests, respectively. The entire slide was scanned manually by a trained observer and the cancer cells were identified based on the fluorescence intensity, size and morphology of the stained cells. Figure 2 shows example images of the cancer cells (the cells with green fluorescence), which were captured on PEN film-coated glass slides. Non-specifically captured WBCs (the cells with red fluorescence) were also found with a certain frequency, as shown in the images. The WBCs were removed from the sample in the next step. We should note that sample staining might degrade the quality of RNA, as reported in literature [20, 21]. However, immunostaining in laser microdissection-based RNA analyses is still commonly used and has shown success [22, 23]. In the case of a rare tumour cell analysis, the use of immunostaining seems an acceptable compromise to identify the tumour cells of interest from the majority of the white blood cells.

**Figure 3.**
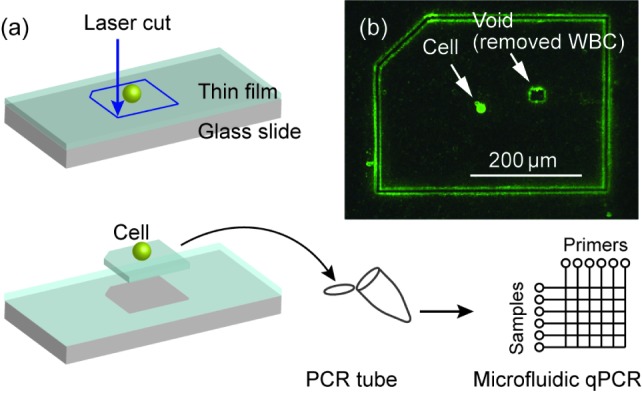
(a) Immunofluorescence-assisted selection of the single cancer cells via a laser microdissection. A portion of the PEN film containing a single cancer cell was cut into a small piece of about ~500 μm. (b) Fluorescence image of a cut film. A white blood cell was removed by laser cutting (see the small square-shaped void in the picture). The film was manually picked up by a needle under a microscopic observation and placed onto the cap of a PCR tube. The side of the cut film containing the cancer cells should have been upside.

Once a cancer cell was located, an area of about 500 μm × 500 μm of the film containing the cell was cut by a focused laser beam (see Figure 3(a)). We used a Zeiss PALM MicroBeam Laser Microdissection. In this step, the contaminating WBCs were identified with the CD45 fluorescent marker through immunofluorescence. The areas containing them were selectively cut out using a Laser Microdissection. The number of WBCs in the 500 μm × 500 μm was typically 3~5 or less. With the resolution of the laser tool (~a few micrometres), most of the WBCs could be avoided or safely removed from the cut portion, eliminating possible contamination from the WBCs that non-specifically remained on the slides after the microfluidic separation. Figure 3(b) shows an example of a cut film, which contained a MCF7 cancer cell. The top left corner of the cut rectangle was trimmed to indicate the film's orientation (upside versus downside). In this example, one WBC was found close to the cancer cell and was removed by laser cutting. This left a void in the cut film. The film was manually picked up by a needle under a microscopic observation and placed onto the adhesive part of a specially designed PCR tube (Zeiss AdhesiveCap tube). The size of the cut film (500 μm × 500 μm) was chosen for an experienced operator to comfortably handle the film. After the film was placed, we conducted a microscopic observation to verify the successful transfer of the cancer cells. We ensured that all of the cancer cells were still visible and that the side of the cut film containing the cancer cells was upside (i.e., the film's trimmed corner was in the top left) in order to directly expose the cells to the reagents. The SEM of the cells transferred in a tube is shown in Figure 7 in the Methods section.

It took a few minutes for each film to be cut and transferred. According to our experience with patient samples [18], the number of CTCs that are found from 7.5mL of blood is mostly between 1 and 5, and very rarely more than 50. In addition, multiple cells are often found in clusters and can be picked up in a single film. In most cases, all of the identified cancer cells were picked up with reasonable effort. With rare cases of more than 1000 cells, we could randomly pick up ~50 cells to conduct the PCR analysis.

### 2.3 RNA Extraction and Reverse Transcription

We used a single cell RNA extraction kit (RNAGEM tissue, ZyGEM Corp. Ltd) to extract mRNA from the sample. To obtain cDNA through reverse transcription, qScriptcDNA-SuperMix (Quanta Biosciences) was used. The cDNA sample was then added with a mixture of primers for target DNA sequences and preamplified by 20-23 cycles before being analysed in the microfluidic qPCR system (BioMark HD System[24], Fluidigm Corporation and StepOne Plus, Applied Biosystems).

We used primers for the gene sequences of UBB (references), ESR1 (for luminal or ER/PR+ cancer cells), HER2 and GRB7 (for HER2+ cancer cells), EPCAM, KRT7, KRT8, KRT18, and KRT19, all of which have been commonly discussed for the purpose of breast cancer diagnosis [7, 25], [26]. The primers were designed by the following method. First, the primer sequences were found from a public resource for PCR primers, PrimerBank website [27]. To prevent the amplification of genomic DNA, two PCR primers (forward and reverse) were designed to span exonexon junctions for the gene transcript of interest. The length of the primers was approximately 20–25 bases and the melting temperature (T_m_) was approximately 60–62°C.

5μl cDNAs were preamplified using 10 μl of 2X Taqman preamp Master Mix (Applied BioSystems Cat# 4391128), 2 μl of 500 nM pooled primer mixture and 3 μl of Nuclease-Free water (Ambion AM9937). The preamplification program was 95 °C for 10 min, 95 °C for 15 s, 60 °C for 4 min for various cycles. Subsequently, the preamplified cDNAs were incubated with 1.6 μl of Exonuclease I (New England Biolabs Cat# M0293), 0.8 μl of Exonuclease I reaction buffer, 5.6μl of water in 37°C for 30 min and inactivated in 80°C for 15 min. The exonuclease treated samples were further diluted 5-fold using TE (TEKnova PN T0224) and the gene expressions were analysed with 48.48 Dynamic Arrays on a BioMark System (Fluidigm).

**Figure 4.**
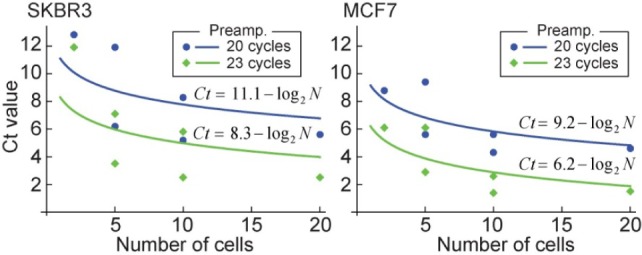
The Ct values for GAPDH with different numbers of cancer cells. The number of cancer cells tested ranged from 2 to 20.

In order to assess the sensitivity of our analysis method, the Ct values of GAPDH for different numbers of SKBR3 and MCF7 cells were compared. For this measurement, the cells were directly prepared on PEN-coated glass slides and stained with FITC. The cells were then observed under a fluorescent microscope and known numbers [2][Bibr bibr3-61822][Bibr bibr4-61822][Bibr bibr5-61822][Bibr bibr6-61822][Bibr bibr7-61822][Bibr bibr8-61822][Bibr bibr9-61822][Bibr bibr10-61822][Bibr bibr11-61822][Bibr bibr14-61822][Bibr bibr13-61822][Bibr bibr14-61822][Bibr bibr15-61822][Bibr bibr16-61822][Bibr bibr17-61822][Bibr bibr18-61822][Bibr bibr19-61822]–[20] of cells were picked up by a laser microdissection. After cDNA was produced from the extracted mRNA, the samples were preamplified by 20 or 23 cycles and analysed by qPCR (StepOne Plus, Applied Biosystems). The result is shown in Figure 4. The curves were found by fitting the theoretically expected formula of *Ct* = A - log_2_*N* to the measured Ct values. It was shown that the Ct values for GAPDH directly related to the number of cells that were tested.

**Figure 5.**
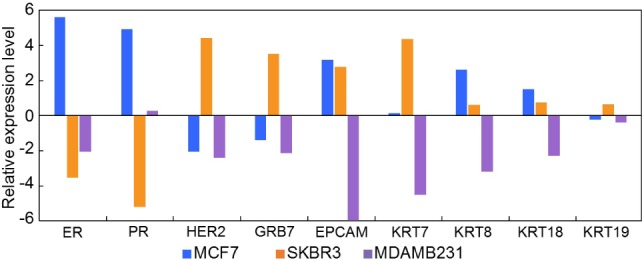
The relative expression values of the reference cancer cells (a few thousand cells) for the primers that were used in this study. The values were scaled by log_2_X and normalized by the whole data set for each primer. We used breast cancer cell lines of MCF7, SKBR3 and MDAMB231 to model ER/PR+, HER2+, and triple negative (TN) breast cancer cells, respectively.

Along with the single cell experiment, the appropriateness of the choice of primers was tested using a qPCR analysis of a few thousand cells for each of the cell lines including MCF7, SKBR3 and MDAMB231. The cells were directly prepared from culture dishes. The Biomark HD system was used in this test. Figure 5 shows the relative expression levels. Expressions of UBB were used as references. The values were scaled by log_2_X (larger, higher expression) and normalized by the whole data set for each primer (i.e., the average expression level of three cell lines for each primer was set to zero). As listed below, the expected characteristics were observed.

- The MCF7 cells (luminal) showed the highest ER and PR expressions
- The SKBR3 (HER2+) cells showed the highest HER2 and GRB7 expressions
- The MDAMB231 cells showed the lowest expressions of EPCAM and KRT

### 2.4 Single Cell PCR Analysis

**Figure 6.**
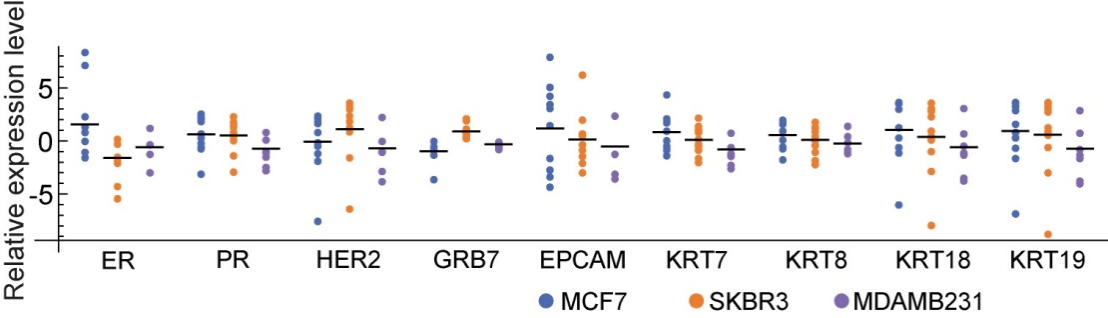
The result of the single cell analysis. The relative expression values for the single cell samples were plotted. The values were scaled by log_2_X and normalized by the whole data set for each primer. For MCF7 and SKBR3, the single cells that were spiked into and captured from blood samples were used. For MDAMB231, the control cells (single cells directly prepared on a glass substrate) were used.

A single cell PCR analysis of cancer cells separated from blood was performed. After the cancer cells were captured, stained and identified on the substrate, from one to five, the cancer cells were picked up by laser cutting for each PCR tube. For both MCF7 and SKBR3, 11 samples were each prepared with the cancer cells spiked into and retrieved from blood, and three were prepared from the control slides (the slides where the cells were directly dropcast). Due to the low expression of EPCAM and cytokeratin, it was much more difficult to separate the MDAMB231 cancer cell from blood than the other two cell lines. For MDAMB231, one sample was prepared from a spiked sample and the other seven samples were made from control slides. The samples were then processed with the Biomark HD system. Figure 6 shows the result. Each combination of sample and primer was duplicated and the average was used. A heat map showing all of the measurements obtained from the raw data is shown in Figure 8 in the Methods section. On average, the measured expression levels coincided with the results that were obtained from the reference cells (a few thousand cells) in Figure 5. Although the trend was less significant for the single cell analysis, most of the primers showed a tendency that was expected from the characteristics of ER/PR+, HER2 and TN cells. However, when we looked at the values from each sample, there were cases where the results contradicted what we had expected. It is worth noting that gene expressions that are measured from a cell population do not necessarily represent the expression levels in single cells [28]. In this study, some primers showed noticeable deviations amongst the samples, while other primers had fewer deviations. Here are two interesting examples:

- The MCF7 cells (typically considered ER positive) obviously showed intense expression levels of ER on average. However, some single cells had lower expression levels than the SKBR3 cells (typically considered ER negative).
- Positive expression levels of GRB7 were only found with the SKBR3 cells, and all of the other cells showed negative values (see also Figure 8 in the Methods section).

These indicate that some of the markers that are usually used to identify types of cancers may not work properly for a single cell analysis. Cell heterogeneity is a critical factor that is relevant to a single cell analysis. The primers that show smaller deviations should be used to supplement an analysis for single cell cancer profiling.

## 3. Conclusion

We have demonstrated a technique for a single cell PCR analysis of CTCs that is based on microfluidic immunomagnetic separation. Small numbers of cancer cells (from one to five) were successfully separated from blood samples and tested in a commercially available microfluidic PCR system. This method used standard glass slides and is compatible with most of the existing microscopic observation techniques. The results showed a good match with the result that was obtained from a few thousand cells. It is also important to note that the single cell analysis showed larger deviations in expression levels, and some markers (e.g., ER, HER2) that are commonly used for cancer cell identification may not represent the characteristics of individual cells. Other primers (e.g., GRB7) showed smaller deviations and may be more suitable for single cell profiling. The results have shown the feasibility of using CTCs as the source of a detailed genomic analysis of tumours or future “liquid biopsy”, which is based on a blood analysis.

## 4. Methods

### 4.1 Single Cell RNA Extraction

**Figure 7.**
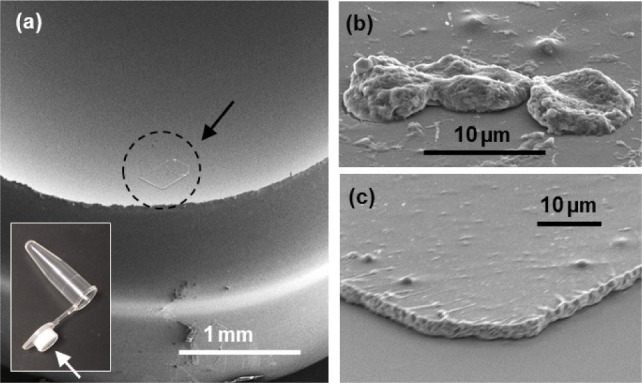
Single cell preparation. (a) A laser cut PEN film was placed onto the adhesive part of the PCR tube. (b) The number of cells that were picked up for each tube ranged from 1 to 5. (c) The film thickness was approximately 3μm. It was viewed with a viewing angle of about 45 degrees.

Figure 7 shows the PCR tube and a cut film that has three cancer cells. After a cut film was placed on the cap of a PCR tube, the steps of an RNA extraction were performed within the cap to reduce the volume of the reagents that were used. The tube was kept upside down during the extraction. First, 0.2 μL of RNA GEM was added onto the cap. The tube was incubated at 75°C for 5 min, and 0.28 μL of TE buffer was added.

### 4.2 Single Cell qPCR Analysis

**Figure 8.**
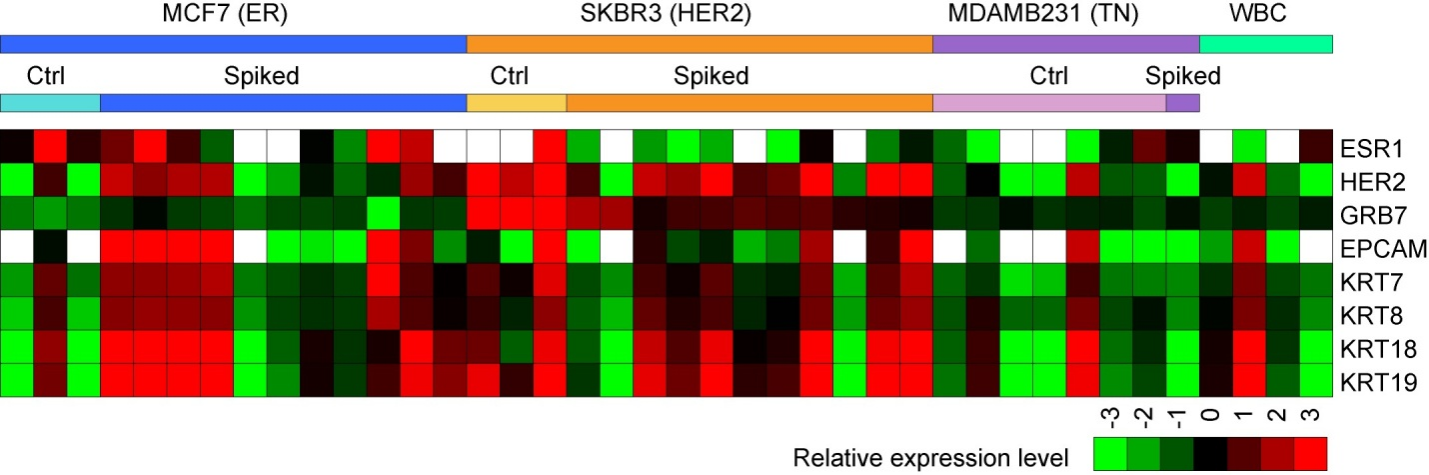
Heat map obtained from 40 samples and nine primers. Each mixture of a sample and a primer was duplicated, and the average was used in the map. Each sample contained 1-5 single cells. The control cells were cells that were directly prepared on a PEN-coated glass slide. The spiked cells were spiked into a blood sample and captured on a PEN-coated glass slide by the immunomagnetic separation. For GRB7, the intense expressions (shown in red) were only found with the SKBR3 cells.

Figure 8 shows a heat map of the measurements with the FluidigmBiomark System. The expression levels of UBB were used as the reference. For each primer, the values were normalized by the average of the whole set of samples. The values lower than −3 or higher than 3 are treated as −3 or 3, respectively. The blank panels mean “undetectable.”

## 5. Compliance with Ethical Research Standards

All of the authors declare no conflict of interest.
